# Oleanolic Acid and Ursolic Acid Induce UGT1A1 Expression in HepG2 Cells by Activating PXR Rather Than CAR

**DOI:** 10.3389/fphar.2019.01111

**Published:** 2019-09-27

**Authors:** Na Yao, Caiwen Zeng, Tao Zhan, Fang He, Mingyi Liu, Fanglan Liu, Hong Zhang, Yuqing Xiong, Chunhua Xia

**Affiliations:** ^1^Clinical Pharmacology Institute, Nanchang University, Nanchang, China; ^2^Laboratory of Translational Medicine and Oncology, Jiangxi Province Cancer Hospital, Nanchang, China; ^3^Pharmacy Department, Jiangxi Province Cancer Hospital, Nanchang, China

**Keywords:** OA, UA, UGT1A1, PXR, CAR

## Abstract

**Background:** Oleanolic acid (OA) and its isomer ursolic acid (UA) have recently emerged as research foci based on their biologic activities. We previously demonstrated that UA can inhibit the activities of UGT1A3 and UGT1A4, and OA inhibits UGT1A3 activity in liver microsomes. However, whether OA and UA affect the expression of UGT1As in HepG2 cells and the underlying regulatory mechanism remain unclear.

**Purpose:** The present study aimed to explore the effect of OA and UA on the expression of UGT1As in HepG2 cells and the regulatory mechanisms on UGT1A1 based on the pregnane X receptor (PXR) and constitutive androstane receptor (CAR) signaling pathways.

**Methods:** We analyzed the effect of OA and UA on UGT1A expression and on the PXR/CAR regulatory pathway in HepG2 cells, hPXR-silenced HepG2 cells, and hCAR-silenced HepG2 cells by Q-PCR, Western blotting, and dual-luciferase reporter gene assays.

**Results:** In HepG2 cells, OA and UA both significantly induced the expression of UGT1A1, UGT1A3, UGT1A4, and UGT1A9 and upregulated the expression of PXR. However, OA and UA did not affect CAR expression. A dual-luciferase reporter assay showed that OA and UA could markedly promote PXR-mediated UGT1A1 luciferase activity, whereas OA and UA did not affect CAR-mediated UGT1A1 luciferase activity. In hPXR-silenced HepG2 cells, OA and UA did not elevate UGT1A1 activity compared to the control group. However, the expression of UGT1A1 in hCAR-silenced HepG2 cells was markedly elevated compared to the control group or with non-silenced HepG2 cells treated with OA (10, 20, and 40 μM) or UA (10, 20, and 40 μM).

**Conclusions:** OA and UA significantly induce the expression of UGT1A1, UGT1A3, UGT1A4, and UGT1A9 in HepG2 cells, and their induction on UGT1A1 is mediated by PXR activation, not CAR.

## Introduction

UDP-glucuronosyltransferases (UGTs) are the major conjugation enzymes of phase II metabolism *in vivo*, which plays a vital role in maintaining the balance of endogenous metabolites (e.g., bile acids, bilirubin, and steroids) and the removal of exogenous metabolites (e.g., drugs, environmental chemicals, and dietary constituents) (Haberkorn et al., 2002; Manevski et al., 2013; Street et al., 2017). In humans, UGTs are divided into two main subfamilies, namely, UGT1 (e.g., UGT1As) and UGT2, based on amino acid sequence identity (Tukey and Strassburg, 2000). Most of the UGTs are expressed in the liver, including UGT1A1, UGT1A3, UGT1A4, UGT1A6, UGT1A9, UGT2B4, UGT2B7, UGT2B10, UGT2B11, UGT2B15, UGT2B17, and UGT2B28, whereas UGT1A7, UGT1A8, UGT1A10, and UGT2A1 are distributed in extrahepatic tissues (Mackenzie et al., 2005; Nakamura et al., 2008).

UGT1A1, which is known as a bilirubin metabolic enzyme, is one of the most concerned UGT isoforms and is involved in the metabolism of various drugs (e.g., etoposide, trafloxacin, and flavonoids) and important endogenous substances (e.g., bilirubin metabolism and irinotecan detoxification) (Wen et al., 2007; Kapitanović et al., 2009; Yeh et al., 2016). Changes in UGT1A1 activity may lead to alterations in drug metabolism and increased risk for clinical drug–drug interactions. For example, a decrease of UGT1A1 activity reduces glucuronidation of SN-38 (a pharmacologically active metabolite of the anticancer drug, irinotecan), leading to an increased risk for the development of severe irinotecan-associated toxicity (Takano and Sugiyama, 2017). Hence, it is important and significant to investigate the regulation of the UGT1A1 gene for the prevention and treatment of bilirubin metabolic disease and clinical drug–drug interactions.

In general, UGTs are regulated by a group of transcription factors, including the constitutive androstane receptor (CAR), pregnane X receptor (PXR), aryl hydrocarbon receptor (AhR), and peroxisome proliferator-activated receptor alpha (PPARa) (Bock, 2010). In a more recent report, the UGT1A1 5′-upstream 290-bp region, which contains multiple CAR-, PXR-, and AhR-response elements, was found to be inducible in response to flavonoids and xenobiotics (Sugatani et al., 2004). Several studies have shown that the expression of bilirubin-detoxifying enzymes and transporters is regulated by the transcription control of the orphan nuclear receptor PXR and CAR, and they confirmed that UGT1A1 is a target gene for PXR and CAR (Maglich et al., 2002; Xie et al., 2003). CAR and PXR are characterized by the presence of a DNA-binding domain (DBD) with two zinc finger motifs and a conserved ligand-binding domain (LBD), which, in addition to the ligand-binding site, contains dimerization motifs and trans-activation domains (Stanley et al., 2006). Both CAR and PXR form heterodimers with retinoid X receptor (RXR) and bind to the enhancer region of target genes, mediating the transcription of target genes (Kliewer et al., 2015). Xie et al. (2003) found that activated PXR and CAR can induce UGT1A1 and UGT1A6 expression *via* strong binding with the elements of DR-3 in the upstream of the promoters for PXR and CAR. In addition, some studies have shown that rifampicin and phenobarbital mediate the transcriptional activity of UGT1A1 *via* the PXR and CAR signal pathway, respectively (Sugatani et al., 2005; Sugatani et al., 2012).

OA and UA are pentacyclic triterpenoid isomers that are widely distributed in various plants. Studies have demonstrated that OA and UA possess several bioactivities, such as hepatoprotection, anti-tumor, and anti-inflammatory bioactivities (Victor et al., 2015; Kashyap et al., 2016). We previously reported that UA is mainly metabolized by UGTs, and UGT1A3 and UGT1A4 are the main enzymes responsible for the glucuronidation of UA in humans (Gao et al., 2016). Additionally, we showed that UA undergoes competitive inhibition with both UGT1A3 and UGT1A4, and OA exhibits mixed competitive inhibition with UGT1A3. *In vivo* and *in vitro* predictions suggest that UA and OA are more likely to interact *in vivo* when co-administered with UGT1A3 or UGT1A4 substrates (Xie et al., 2017). However, whether UA and OA influence the expression of UGTs in HepG2 cells and whether the regulation is associated to the PXR and CAR signaling pathway remain unclear.

Therefore, the purpose of this study was to investigate the effect of OA and UA on the expression of UGT1A in HepG2 cells and the underlying regulatory mechanisms based on the PXR and CAR signaling pathway using HepG2 cells, hPXR-silenced, or hCAR-silenced HepG2 cells.

## Materials and Methods

### Reagents and Chemicals

OA (purity ≥ 94.9%) and UA (purity ≥ 98.0%) were purchased from the National Institute for the Control of Pharmaceutical and Biological Products (Beijing, China). Rifampicin (RIF, purity ≥ 97.0%) was purchased from Sigma-Aldrich (St. Louis, MO, USA). Six-(4-chlorophenyl)imidazo (2,1-b)(1,3) thiazole-5-carbaldehyde O-(3,4-dichlorobenzyl) oxime (CITCO, purity 98.0%) was purchased from APExBIO Company (Beijing, China). Dulbecco’s modified Eagle’s Medium (DMEM), fetal bovine serum (FBS), and other cell culture reagents were procured from Hyclone (Logan, UT, USA). Primers for the UGT1A1, UGTA3, UGT1A4, UGT1A9, PXR, CAR, and GAPDH genes for quantitative real-time polymerase chain reaction (Q-PCR) were commercially synthesized by Sangon Biotech Co., Ltd (Shanghai, China). Antibodies used for immunoblot detection, including anti-human UGT1A1, UGT1A3, UGT1A4, and UGT1A9 antibody were all from Abcam (Cambridge, MA, USA). Rabbit polyclonal PXR and CAR antibody were obtained from Proteintech (Chicago, IL, USA). Mouse polyclonal anti-β-actin antibody was obtained from Sigma Co., Ltd (St. Louis, MO, USA). Mouse polyclonal anti-Lamin B1 antibody was obtained from Santa Cruz biotechnology Co., Ltd (Santa Cruz, CA, USA). The dual-luciferase reporter assay system was purchased from Promega (Madison, WI, USA). The SuperFectin II DNA transfection reagent was purchased from PUFEI Biology (Shanghai, China). All of the other chemicals and solvents were of the highest grade or analytical grade commercially available.

### Plasmids

The UGT1A1 reporter gene plasmid containing UGT1A1 5′-upstream 290-bp distal enhancer module (−3,483/−3,194) with the PXR-, AhR-, and CAR-binding sites was constructed by Maijie Biotechnology Co., Ltd (Nantong, Jiangsu, China) (Sugatani et al., 2004b). The reporter gene was from human genomic DNA and cloned into the pGL3-promoter vector (Promega), namely, pGL3-UGT1A1. The expression plasmid pcDNA3.1 (+)-hCAR containing the full-length human CAR, expression plasmid pcDNA3.1 (+)-hPXR containing the full-length human PXR, pGL3-Basic, and pcDNA3.1 (+) were provided by Maijie Biotechnology Co., Ltd (Nantong, Jiangsu, China). The pRL-TK renilla luciferase plasmid was obtained from Promega and used to normalize firefly luciferase activities.

### Cell Culture and Treatment

The HepG2 cells were obtained from Boster Biological Technology Co., Ltd (Wuhan, China). In our study, hPXR-silenced HepG2 and hCAR-silenced HepG2 cell models were successfully constructed by respectively transfecting shPXR and shCAR plasmids using the SuperFectin II DNA transfection reagent (PUFEI Biology) following the manufacturer’s instructions. The HepG2 cells were cultured in Dulbecco’s modified Eagle’s medium (DMEM) with 10% FBS at 37°C in a humidified 5% CO_2_ atmosphere. Cell passages were performed with 0.25% trypsin-EDTA (Gibco, Life Technologies) when cells reached 80–90% confluency. For the treatments, OA, UA, RIF, or CITCO was dissolved in dimethyl sulfoxide (DMSO) for the final stock solution concentrations of 20, 20, 50, and 50 mM, respectively and diluted with DMEM containing 10% FBS. The final concentration of DMSO never exceeded 0.1% (v/v) in the medium.

### Quantitative Real-Time Polymerase Chain Reaction Analysis

Total RNA was extracted from HepG2 cells using TRIzol (Invitrogen, Grand Island, NY, USA) according to the manufacturer’s instructions. Total RNA with an OD_260_/OD_280_ between 1.8 and 2.0 was used for qRT-PCR. Approximately 2 μg of total RNA from each sample was reversely transcribed into cDNA using a PrimeScript RT reagent kit (TaKaRa Biotech, Kyoto, Japan). Real-time PCR was performed using a PrimeScript™ RT-PCR kit (TaKaRa Biotech, Kyoto, Japan) according to the manufacturer’s instruction in a Thermal Cycler Dice Real Time System. All of the samples were quantified using a comparative threshold PCR cycle (C_t_) method for relative quantification of gene expression, normalized to that of glyceraldehyde-3-phosphate dehydrogenase (GAPDH). The primer sequences are listed in **Table 1**.

**Table 1:** Primer sequences used for qRT-PCR.

Gene	Forward primer (5′→3′)	Reverse primer (5′→3′)
UGT1A1	CCTTGCCTCAGAATTCCTTC	ATTGATCCCAAAGAGAAAACCAC
UGT1A3	TGTTGAACAATATGTCTTTGGTCT	CACAGGACTGTCTGAGGGATTTT
UGT1A4	CCTGCTGTGTTTTTTTGGAGGT	ATTGATCCCAAAGAGAAAACCAC
UGT1A9	GAACATTTATTATGCCACCG	ATTGATCCCAAAGAGAAAACCAC
PXR	TGTTCGGCATCACAGGTAGC	GGCTCTTGGCAGTGTCCATC
CAR	GCTGGCATGAGGAAAGACAT	CGGATCAGCTCTTCTTGCTC
GAPDH	CAGGAGGCATTGCTGATGAT	GAAGGCTGGGGCTCATTT

### Western Blotting Analysis

Total protein fractions were extracted using the RIPA lysis buffer (Solarbio Co., Ltd, Beijing), and nuclear protein fractions were extracted from HepG2 cells, hPXR-silenced HepG2 cells and hCAR-silenced HepG2 cells using a nuclear extraction kit (Active Motif, Carlsbad, CA, USA) according to the manufacturer’s instruction. Protein concentrations were measured with bicinchoninic acid assay (BCA) protein assay (Vazyme Biotech Co., Ltd., Nanjing, China). Protein samples (50 μg) were subjected to 10% sodium dodecyl sulfate-polyacrylamide gel electrophoresis (SDS-PAGE) and electrophoretically transferred onto a polyvinylidene fluoride (PVDF) membrane (EMD Millipore, Bedford, MA, USA). Subsequently, the membranes were blocked with 5% skim milk for 2 h and then incubated at 4°C overnight with primary antibodies, including UGT1A1 (1:1,000; ab194697, Abcam, Cambridge, MA, USA), UGT1A3 (1:2,000; ab57400, Abcam), UGT1A4 (1:1,000; ab192424, Abcam), UGT1A9 (1:1,000; ab96214, Abcam), PXR (1:2,000; Proteintech, Chicago, IL, USA), CAR (1:2,000; Proteintech, Chicago, IL, USA), β-actin (1:5,000; Sigma Co., Ltd., USA), and Lamin B1 (1:5,000; Santa Cruz, CA, USA). Then, the membrane was incubated for 1 h with secondary horseradish peroxidase-conjugated anti-rabbit or anti-rat IgG antibody (1:5,000; Santa Cruz, CA, USA). Subsequently, signals were detected by SuperSignal West Dura (Pierce, Rockford, IL) using a Bio-Rad ChemiDoc XRS imaging system (Bio-Rad Laboratories). Densitometry analysis was performed using Image Lab software (Bio-Rad Laboratories). Mouse polyclonal anti-β-actin and mouse polyclonal anti-Lamin B1 antibody were used as loading control for total proteins and nuclear proteins, respectively.

### Transient Co-Transfection and Luciferase Reporter Assays

HepG2 cells were cultured in high-glucose (4.5 g/L) DMEM with 10% FBS. The cells were cultured in a 24-well plate to 70–80% confluency before transfection. At 1 h before transfection, DMEM with 10% FBS was replaced with fresh Dulbecco’s Modified Eagle Medium (DMEM). The HepG2 cells were transfected with plasmids using SuperFectin transfection reagent according to the manufacturer’s instructions. Each well was incubated with 500 ng of mixed plasmid DNA for 5 h. Empty expression vectors were added to equalize the total amount of plasmid DNA transfected in each assay. For the PXR transactivation assay, each well contains 320 ng of pGL3-UGT1A1-luc/pGL3-Basic, 160 ng of pcDNA 3.1 (+)-hPXR/pcDNA 3.1 (+) and 20 ng of pRL-TK renilla luciferase plasmid. For CAR transactivation assay, each well contained 384 ng of pGL3-UGT1A1-luc/pGL3-Basic, 96 ng of pcDNA 3.1 (+)-hCAR/pcDNA3.1 (+), and 20 ng of pRL-TK renilla luciferase plasmid. After 5 h of transfection, the HepG2 cells were treated with OA (10, 20, and 40 μM), UA (10, 20, and 40 μM) and respective positive agonist CITCO (10 μM) or RIF (10 μM) for 24 h (Tolson and Wang, 2010). DMSO (0.1%) was used as the negative control. Subsequently, the cells were lyzed in the passive lysis buffer (1× PLB) (Promega). The total cell lysates were carefully harvested into the pre-labeled 1.5 ml Eppendorf tubes and then quickly centrifuged at 10,000 rpm for 30 s. The activities of the firefly and Renilla luciferases were assessed using the Dual-Luciferase Reporter Assay System (Promega). The Renilla luciferase activity was used as an internal control. The firefly luciferase activity was normalized against the Renilla luciferase activity, and the ratios of normalized luciferase activity from each tested extract over DMSO treatment were served as relative luciferase activity or fold of induction. Each experiment was repeated three times in triplicate.

### RNA Interference

ShRNA against human CAR (hCAR shRNA) and negative control non-silencing RNA (hCAR shNC) were purchased from the Maijie Biotechnology Co., Ltd. (Nantong, Jiangsu, China). ShRNA against human PXR (hPXR shRNA) and negative control non-silencing RNA (hPXR shNC) were obtained from Novobio Biotechnology Co., Ltd. (Shanghai, China). The target sequence of hCAR shRNA was as follows: GCAUGAGGAAAGACAUGAU. The target sequence of hCAR shNC was as follows: TTCTCC​GAACGTGTCACGT. The target sequence of hPXR shRNA was as follows: Forward 5′-CACCGGAGGTGAGACCCAAAGAAA​GCGAAC TTTCTTTGGGTCTCACCTCC-3′ and Reverse 5′-AAAAGGAGGTGAGACCC AAAGAAAGTTCGCTTTC​TTTGGGTCTCACCTCC-3′. The target sequence of hPXR shNC was as follows: Forward 5′-CACCGCTACACAAATCA GCGATTTCGAAAAATCGCTGATTTGTGTAG-3′ and Reverse 5′-AAAACTAC ACAAATCAGCGATTTTTCGAAAT​CGCTGATTTGTGTAGC-3′. The hCAR shRNA, hCAR shNC, hPXR shRNA, and hPXR shNC were all confirmed by sequencing. The HepG2 cells were seeded into 6-well or 12-well plates for 24 h and transfected with hCAR shRNA, hCAR shNC, hPXR shRNA, or hPXR shNC using SuperFectin transfection reagent according to the manufacturer’s instructions. Knock-down efﬁciency of shRNAs was determined by Western blotting and real-time Q-PCR analysis as earlier described. After the success of CAR or PXR gene knock-out, the HepG2 cells were treated with OA (10, 20, and 40 μM), UA (10, 20, and 40 μM) and respective positive agonist CITCO (10 μM) or RIF (10 μM). DMSO (0.1%) was used as the negative control. Subsequently, Western blotting and real-time Q-PCR analysis were performed.

### Statistical Analysis

All of the experiments were repeated at least three times, and the data are expressed as the mean ± standard deviation (SD). Statistically significant differences were assessed using one-way analysis of variance (ANOVA) followed by a *post hoc* Dunnett’s test or Student’s t-test where appropriate. Values of P < 0.05 were considered to be statistically significant: *P < 0.05, **P < 0.01, and ***P < 0.001.

## Results

### Effect of OA and UA on UGT1As, PXR, and CAR mRNA and Protein Levels in HepG2 Cells

**Figure 1:**
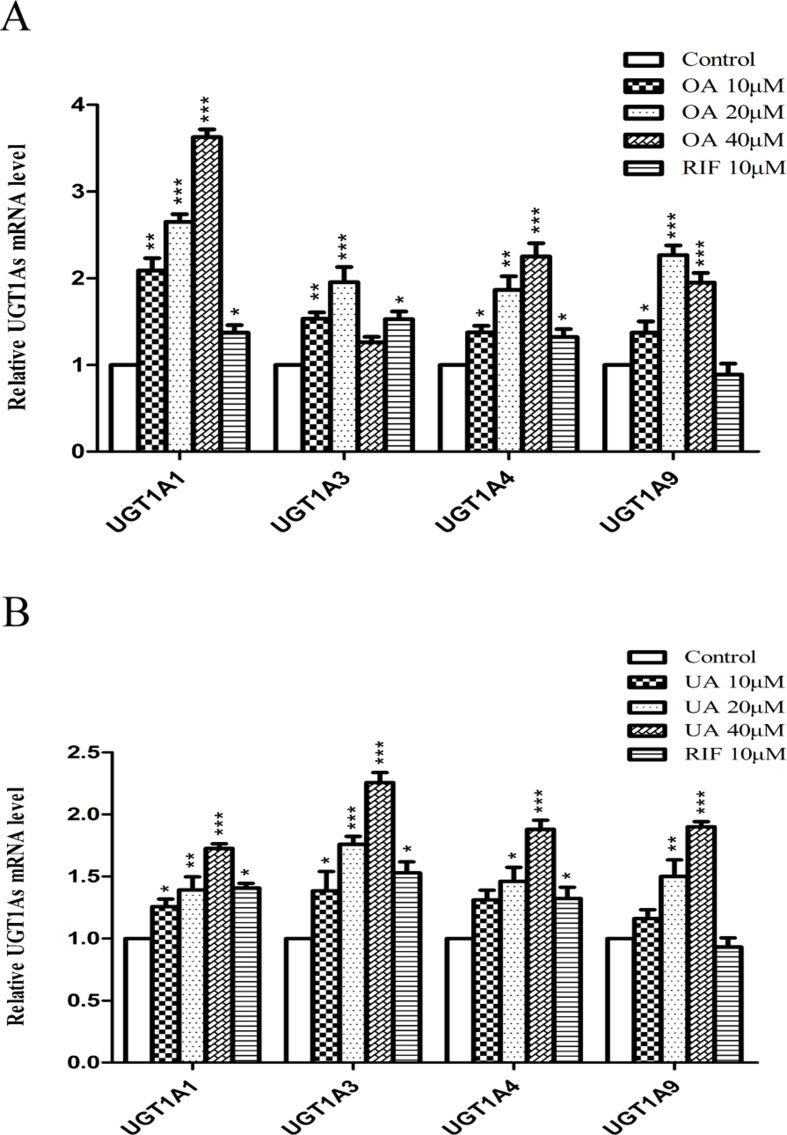
Effect of OA and UA on UGT1A mRNAs in HepG2 cells. The effect of OA (10, 20, and 40 μM) **(A)** and UA (10, 20, and 40 μM) **(B)** on UGT1As (UGT1A1, UGT1A3, UGT1A4, and UGT1A9) mRNA expression in HepG2 cells was quantified by real-time Q-PCR analysis, normalized to GAPDH, and compared to the control group. DMSO (0.1%) was used as the negative control. RIF (10 μM) was used as the positive controls. The data are expressed as the mean ± SD of triplicate independent experiments (*p < 0.05, **p < 0.01, ***p < 0.001).

**Figure 2:**
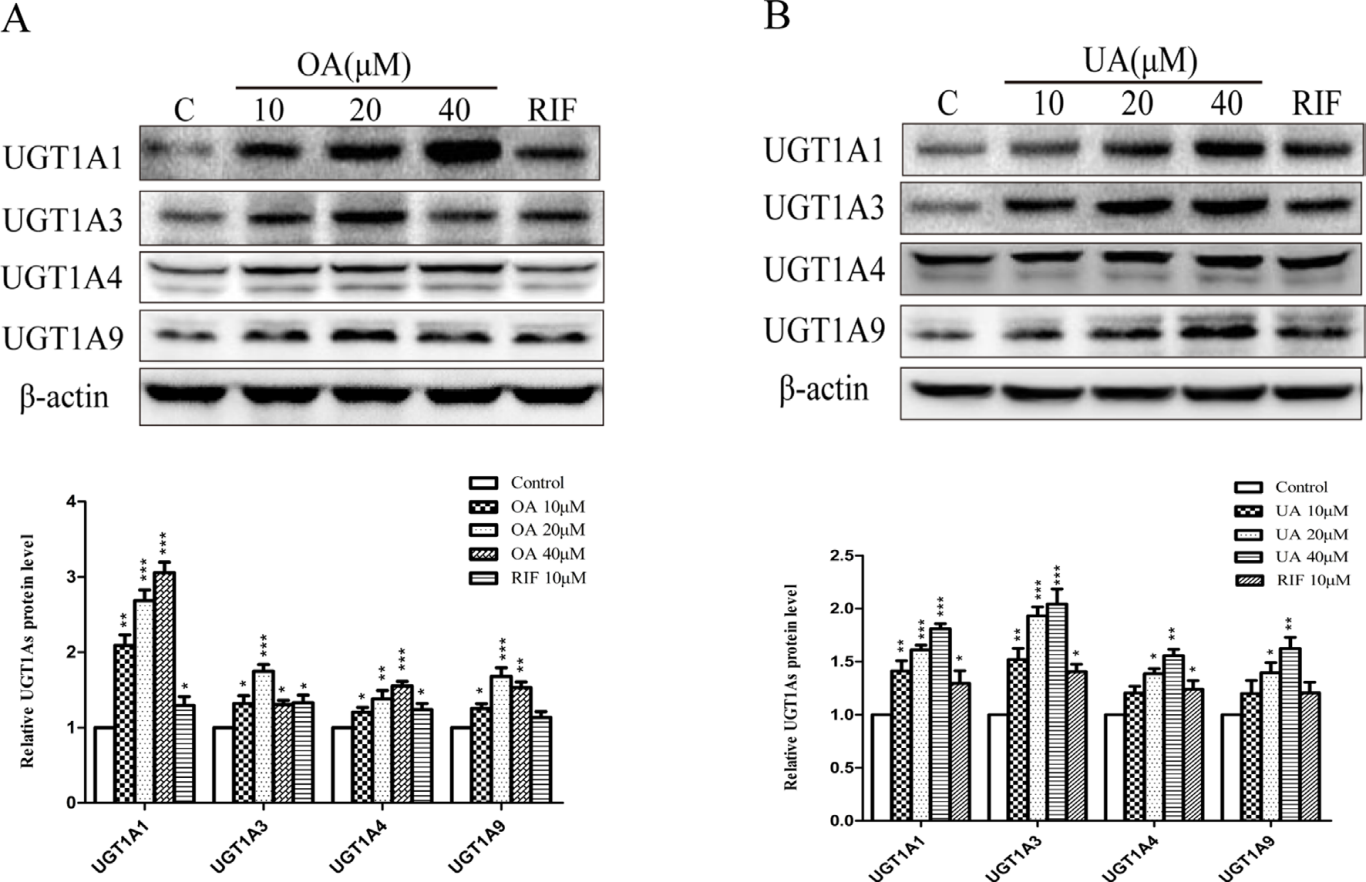
Effect of OA and UA on UGT1A proteins in HepG2 cells. The effect of OA (10, 20, and 40 μM) **(A)** and UA (10, 20, and 40 μM) **(B)** on UGT1As (UGT1A1, UGT1A3, UGT1A4, and UGT1A9) protein expression in HepG2 cells was quantified by Western blotting analysis, normalized to β-actin, and compared to the control group. DMSO (0.1%) was used as negative control. RIF (10 μM) was used as the positive control. The data are expressed as the mean ± SD of triplicate independent experiments (*p < 0.05, **p < 0.01, ***p < 0.001).

To explore the effect of OA and UA on UGT1As, PXR, and CAR, we determined the mRNA and protein levels of UGT1As (UGT1A1, UGT1A3, UGT1A4, and UGT1A9), intranuclear PXR and CAR in HepG2 cells treated with OA (10, 20, and 40 μM), UA (10, 20, and 40 μM), CITCO (10 μM) and RIF (10 μM) for 24 h using real-time Q-PCR and Western blotting assays. The results showed that OA (10, 20, and 40 μM) significantly upregulated UGT1A1 and UGT1A4 mRNA and protein levels in a concentration-dependent manner compared to the control group in HepG2 cells. Similarly, UGT1A3 and UGT1A9 mRNA and protein expression were significantly induced in HepG2 cells treated with OA. Specifically, 20 μM OA exhibited a highest inductive effect on UGT1A3 and UGT1A9. However, UA (10, 20, and 40 μM) significantly promoted UGT1A1, UGT1A3, UGT1A4, and UGT1A9 mRNA and protein expression in a concentration-dependent manner (**Figures 1** and **2**).

**Figure 3:**
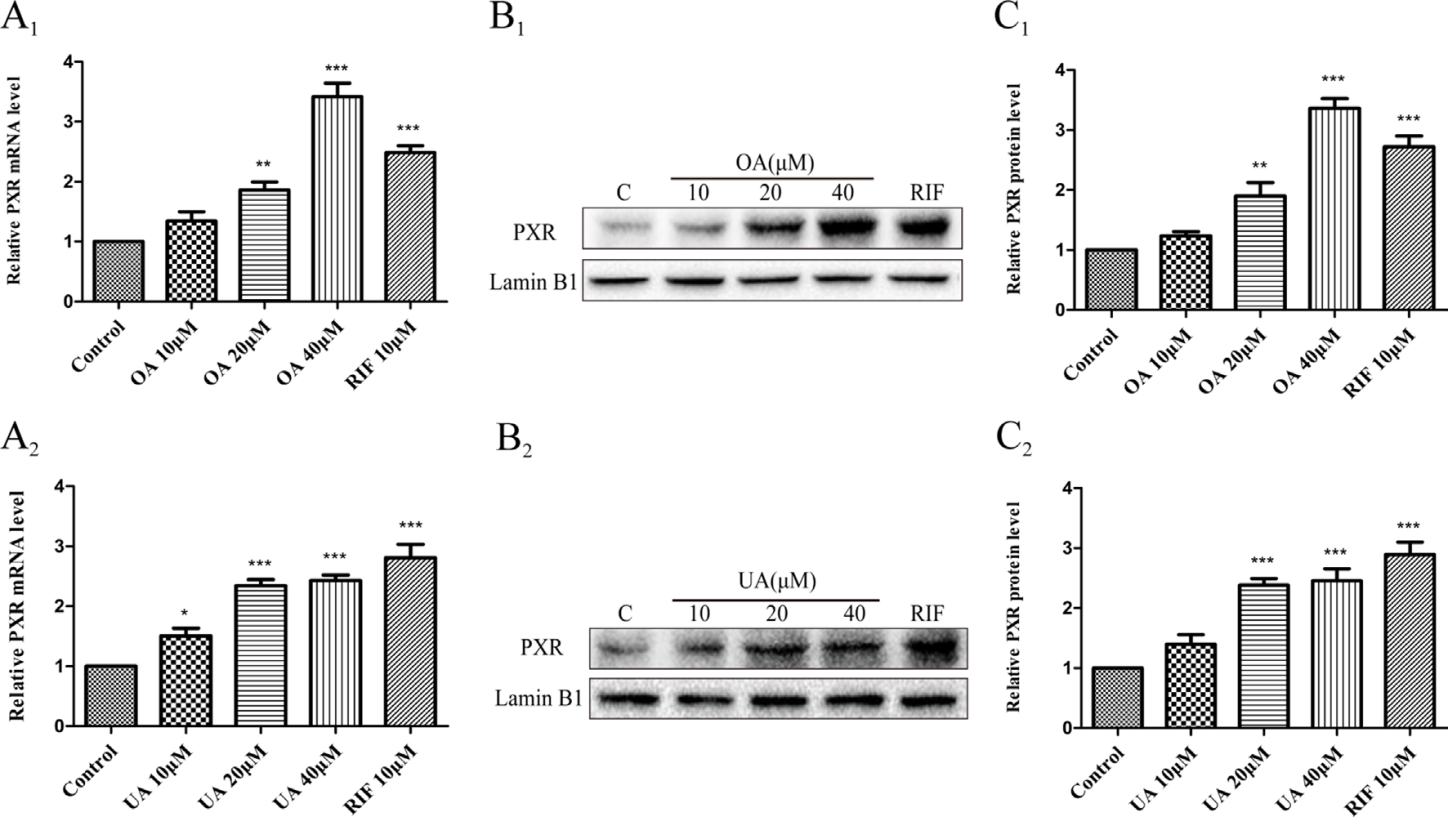
Effect of OA and UA on PXR mRNA and protein in HepG2 cells. The HepG2 cells were treated with OA (10, 20, and 40 μM) **(A** **_1_**–**C** **_1_** **)** and UA (10, 20, and 40 μM) **(A** **_2_**–**C** **_2_** **)** for 24 h. The effect of OA and UA on PXR mRNA and protein expression in HepG2 cells was measured by real-time Q-PCR analysis **(A)** and Western blotting analysis **(B**–**C)**.The determination of mRNA and nuclear protein was normalized to GAPDH and Lamin B1, respectively. DMSO (0.1%) was used as the negative control. RIF (10 μM) was used as the positive control. The data are expressed as the mean ± SD of triplicate independent experiments (*p < 0.05, **p < 0.01, ***p < 0.001).

**Figure 4:**
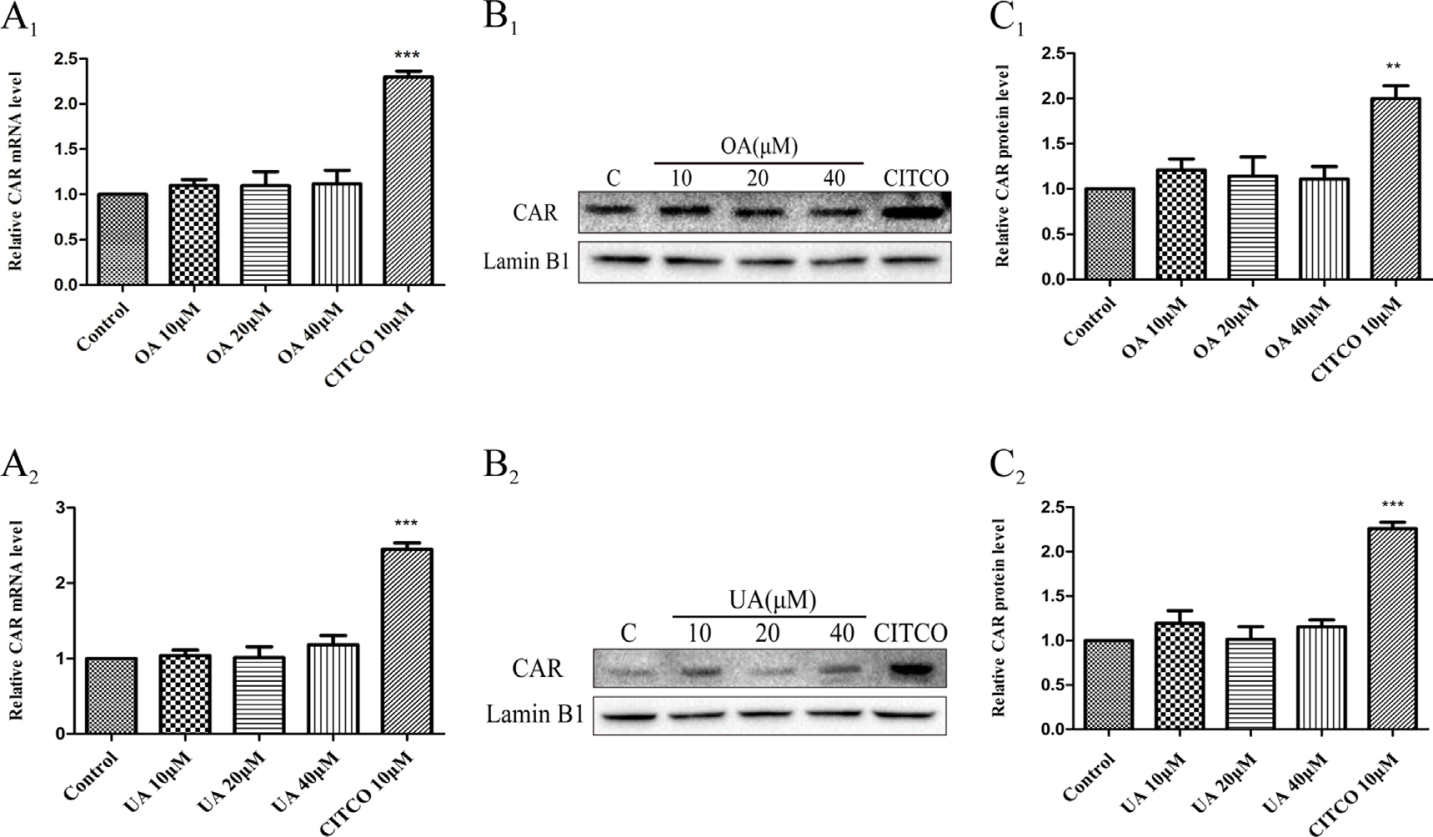
Effect of OA and UA on CAR mRNA and protein in HepG2 cells. The HepG2 cells were treated with OA (10, 20, and 40 μM) **(A** **_1_**–**C** **_1_** **)** and UA (10, 20, and 40 μM) **(A** **_2_**–**C** **_2_** **)** for 24 h. Effect of OA and UA on CAR mRNA and protein expression in HepG2 cells was measured by real-time Q-PCR analysis **(A)** and Western blotting analysis **(B**–**C)**. The determination of mRNA and nuclear protein was normalized to GAPDH and Lamin B1, respectively. DMSO (0.1%) was used as the negative control. CITCO (10 μM) was used as the positive control. The data are expressed as the mean ± SD of triplicate independent experiments (**p < 0.01, ***p < 0.001).

Some studies have indicated that PXR and CAR were localized to the cytoplasm and are translocated into the nucleus after ligand treatment (Kliewer et al., 2015). Thus, the nuclear fractions were extracted from HepG2 cells and then used to explore nuclear translocation of PXR and CAR. The results showed that PXR mRNA and intranuclear protein expression were induced in a concentration-dependent manner in HepG2 cells when treated with OA (10, 20, and 40 μM) and UA (10, 20, and 40 μM) (**Figure 3**). However, no detectable effect on CAR mRNA and protein expression was observed in HepG2 cells treated with OA (10, 20, and 40 μM) and UA (10, 20, and 40 μM) (**Figure 4**). The induction trend of UGT1As elicited by OA/UA was similar to that of PXR, which indicated that PXR may regulate the induction of UGT1As in HepG2 cells treated with OA and UA. To further confirm the underlying regulation mechanism of OA and UA on UGT1As, UGT1A1 was selected as the research objective in the subsequent study.

### Effect of OA and UA on the UGT1A1 Reporter Gene in HepG2 Cells Transiently Transfected With hPXR or hCAR

**Figure 5:**
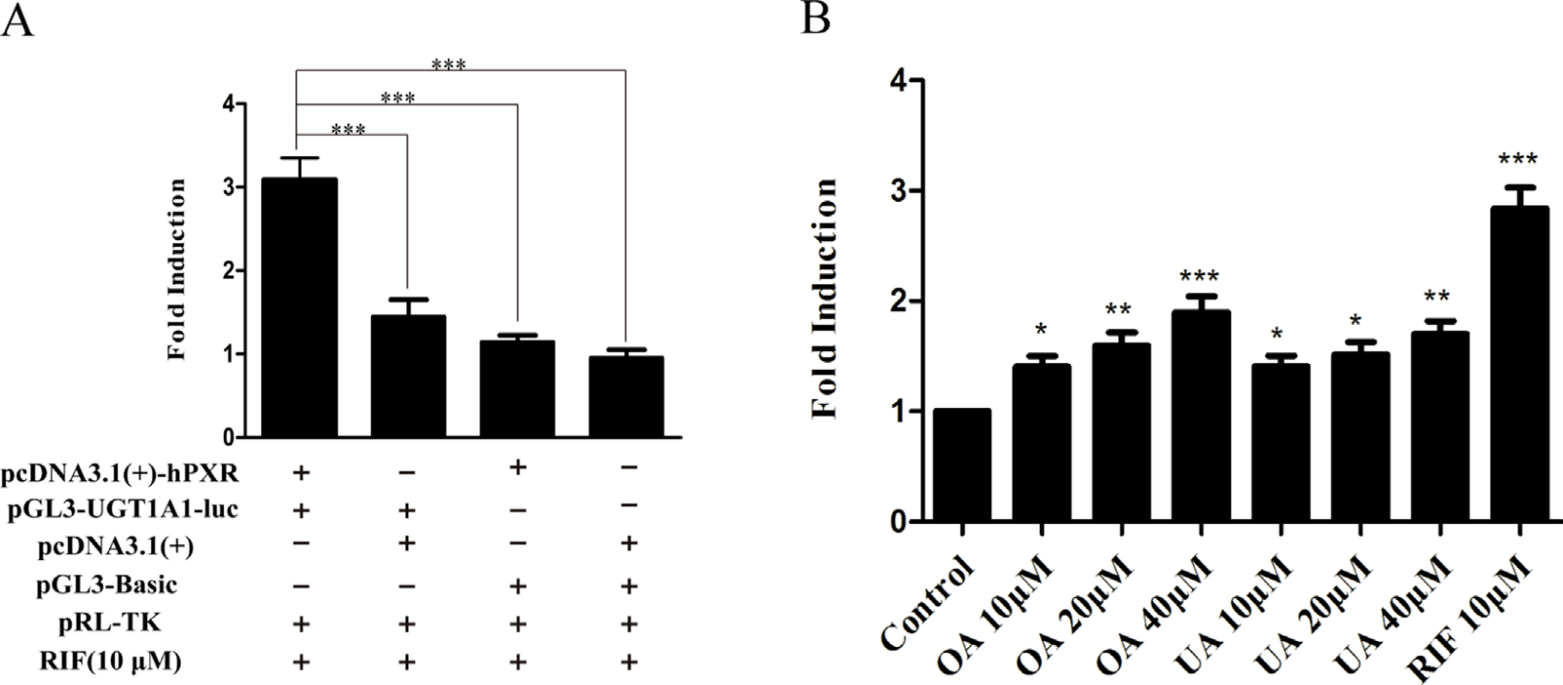
Effect of OA and UA on the UGT1A1 reporter gene in HepG2 cells transiently transfected with hPXR. The HepG2 cells were transfected with pcDNA 3.1 (+)-hPXR, pcDNA 3.1 (+), pGL3-UGT1A1-luc, and/or pGL3-Basic, together with pRL-TK control vector. After transfection for 24 h, the transfected HepG2 cells were cultured with RIF (10 μM) or vehicle DMSO (0.1%) for an additional 24 h **(A)**. The HepG2 cells were transiently co-transfected with pcDNA 3.1 (+)-hPXR expression vector, pGL3-UGT1A1-luc reporter vector, and pRL-TK control vector. After transfection for 24 h, the transfected HepG2 cells were cultured with OA (10, 20, and 40 μM), UA (10, 20, and 40 μM), RIF (10 μM), or DMSO (0.1%) for an additional 24 h **(B)**. Luciferase activity was measured using the dual-luciferase reporter assay system. The transfection efficiency was normalized against renilla luciferase activity from the co-transfected pRL-TK vector. DMSO (0.1%) was used as the negative control. RIF (10 μM) was used as the positive control. The data are expressed as the mean ± SD of triplicate independent experiments (*p < 0.05, **p < 0.01, ***p < 0.001).

**Figure 6:**
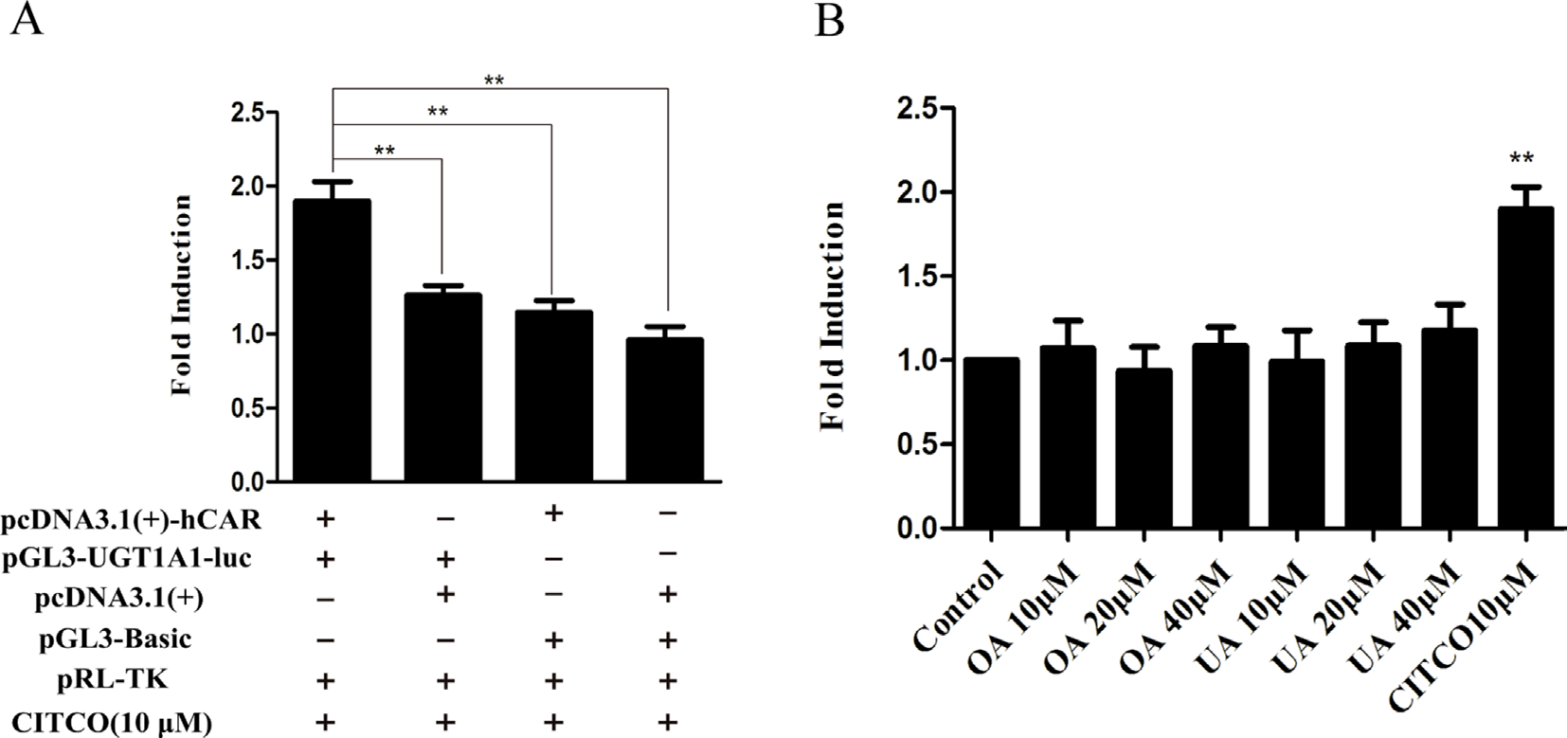
Effect of OA and UA on the UGT1A1 reporter gene in HepG2 cells transiently transfected with hCAR. The HepG2 cells were transfected with pcDNA 3.1 (+)-hCAR, pcDNA 3.1 (+), pGL3-UGT1A1-luc, and/or pGL3-Basic, together with pRL-TK control vector. After transfection for 24 h, the transfected HepG2 cells were cultured with CITCO (10 μM) or vehicle DMSO (0.1%) for an additional 24 h **(A)**. The HepG2 cells were transiently co-transfected with pcDNA 3.1 (+)-hCAR expression vector, pGL3-UGT1A1-luc reporter vector, and pRL-TK control vector. After transfection for 24 h, the transfected HepG2 cells were cultured with OA (10, 20, and 40 μM), UA (10, 20, and 40 μM), CITCO (10 μM), or DMSO (0.1%) for an additional 24 h **(B)**. Luciferase activity was measured using the dual-luciferase reporter assay system. The transfection efficiency was normalized against renilla luciferase activity from the co-transfected pRL-TK vector. DMSO (0.1%) was used as the negative control. CITCO (10 μM) was used as the positive control. The data are expressed as the mean ± SD of triplicate independent experiments (**p < 0.01).

To explore whether PXR and CAR take part in the regulation of UGT1A1 in the presence of UA and OA, we successfully established the PXR or CAR-mediated UGT1A1 reporter gene in HepG2 cells by transient co-transfection (**Figures 5** and **6**). The data on how to calculate the fold induction are shown in **Tables 2** and **3**. **Figure 5A** shows that RIF (10 μM), which was used as the positive control (Tolson and Wang, 2010), significantly increased the PXR-mediated UGT1A1 luciferase activity by 2.83-fold. OA induced the PXR-mediated UGT1A1 luciferase activity by 1.40-fold (10 μM), 1.59-fold (20 μM) and 1.89-fold (40 μM), respectively, while UA induced the PXR-mediated UGT1A1 luciferase activity by 1.40-fold (10 μM), 1.51-fold (20 μM), and 1.70-fold (10 μM), respectively (**Figure 5B**). CITCO (10 μM), which was used as positive control (Tolson and Wang, 2010), significantly increased the CAR-mediated UGT1A1 luciferase activity by 1.90-fold (**Figure 6A**). However, OA (10, 20, and 40 μM) and UA (10, 20, and 40 μM) had almost no significant impact on the CAR-mediated UGT1A1 luciferase activity (**Figure 6B**).

**Table 2:** The relative luciferase activity of PXR-mediated UGT1A1 reporter gene (Mean ± SD, n = 3).

Component	Group		
	Control	RIF (10 μM)	Fold induction
pcDNA3.1(+)-hPXR+pGL3-UGT1A1-Luc+pRL-TK	0.101 ± 0.013	0.311 ± 0.034	3.087 ± 0.150
pcDNA3.1(+)+pGL3-UGT1A1+pRL-TK	0.094 ± 0.007	0.137 ± 0.018	1.451 ± 0.103
pcDNA3.1(+)-hPXR+pGL3-Basic+pRL-TK	0.083 ± 0.010	0.094 ± 0.014	1.135 ± 0.046
pcDNA3.1(+)+pGL3-Basic+pRL-TK	0.078 ± 0.012	0.075 ± 0.010	0.967 ± 0.052

**Table 3:** The relative luciferase activity of CAR-mediated UGT1A1 reporter gene (Mean ± SD, n = 3).

Component	Group		
	Control	CITCO (10 μM)	Fold induction
pcDNA3.1(+)-hCAR+pGL3-UGT1A1-Luc+pRL-TK	0.099 ± 0.010	0.189 ± 0.028	1.899 ± 0.093
pcDNA3.1(+)+pGL3-UGT1A1+pRL-TK	0.096 ± 0.008	0.121 ± 0.015	1.262 ± 0.047
pcDNA3.1(+)-hCAR+pGL3-Basic+pRL-TK	0.075 ± 0.007	0.086 ± 0.012	1.148 ± 0.056

### Effect of OA and UA on UGT1A1 mRNA and Protein Levels in hPXR-Silenced HepG2 Cells

**Figure 7:**
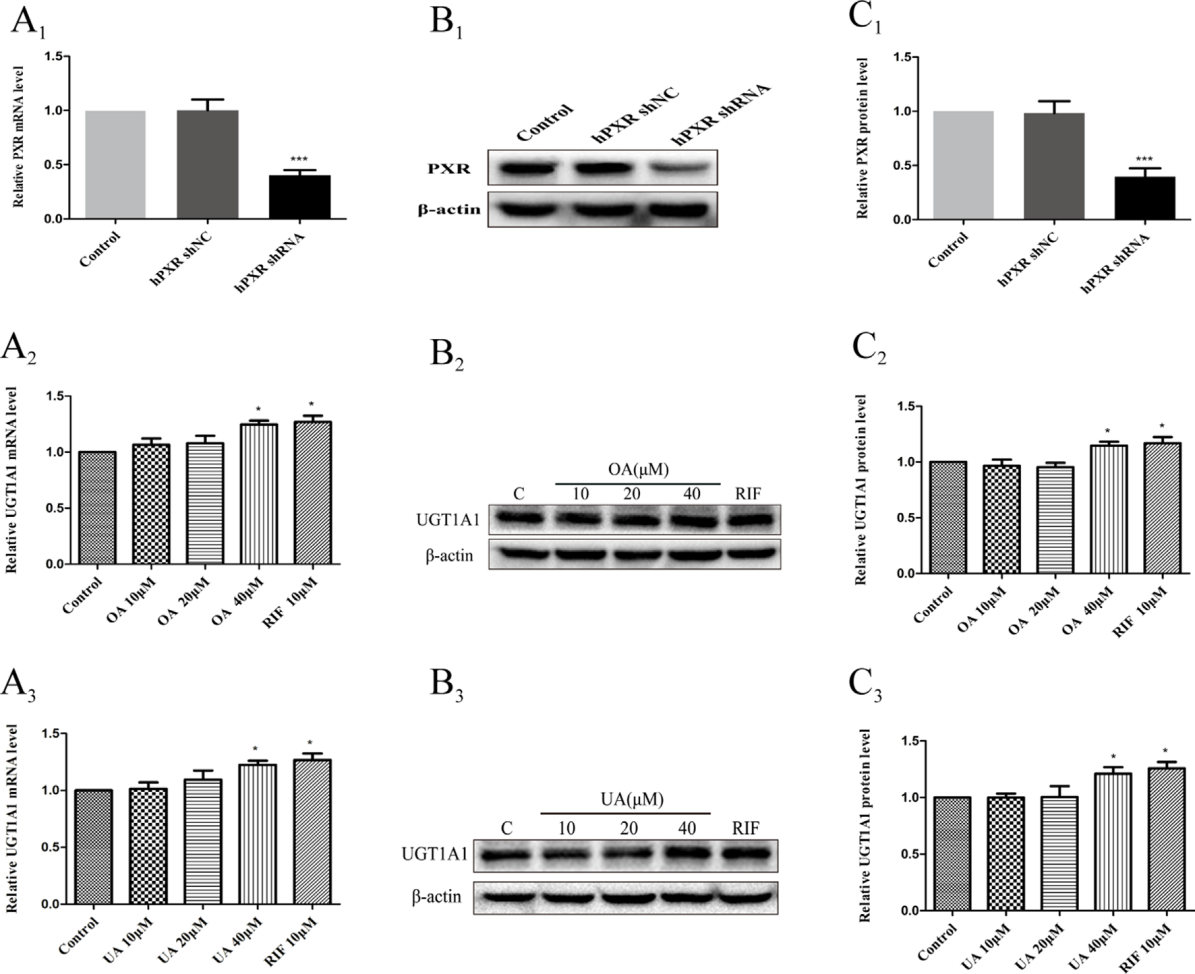
Effect of OA and UA on UGT1A1 mRNA and protein in hPXR-silenced HepG2 cells. PXR mRNA and protein expression in HepG2 cells transfected with hPXR shRNA or hPXR shNC were determined by real-time Q-PCR analysis **(A** **_1_** **)** and Western blotting analysis **(B** **_1_**–**C** **_1_** **)**, respectively. The HepG2 cells transfected with hPXR shRNA were treated with OA (10, 20, and 40 μM) **(A** **_2_**–**C** **_2_** **)** and UA (10, 20, and 40 μM) **(A** **_3_**–**C** **_3_** **)** for 24 h. UGT1A1 mRNA and protein expression in hPXR-silenced HepG2 cells treated with OA and UA were determined by real-time Q-PCR analysis **(A)** and Western blotting analysis **(B**, **C)**, respectively. The determination of mRNA and nuclear protein was normalized to GAPDH and β-actin, respectively. DMSO (0.1%) was used as the negative control. RIF (10 μM) was used as the positive control. The data are expressed as the mean ± SD of triplicate independent experiments (*p < 0.05, ***p < 0.001).

To further confirm that OA and UA may induce PXR-mediated UGT1A1 expression in HepG2 cells, we successfully constructed an hPXR-silenced HepG2 cell model. **Figure 7A1** shows that the expression of PXR mRNA in hPXR-silenced HepG2 cells was downregulated by 69% compared to the control group, and PXR protein was also markedly downregulated by 61% (**Figures 7B1, C1**).

Subsequently, we examined the effects of OA and UA on the UGT1A1 mRNA and protein expression in hPXR-silenced HepG2 cells (**Figures 7A2–C2** and **Figures 7A3–C3**). Compared to the control group (DMSO), RIF (10 μM), OA (40 μM), and UA (40 μM) exhibited a weak induction on UGT1A1 mRNA and protein expression in hPXR-silenced HepG2 cells, whereas treatment with OA (10 and 20 μM) and UA (10 and 20 μM) did not influence the expression of UGT1A1. Moreover, UGT1A1 mRNA and protein expression in hPXR-silenced HepG2 cells treated with OA (10, 20, and 40 μM) and UA (10, 20, and 40 μM) significantly decreased compared to the non-silenced HepG2 cells. The above results indicated that the induction of OA and UA on UGT1A1 was mainly mediated by PXR.

### Effect of OA and UA on UGT1A1 mRNA and Protein Levels in hCAR-Silenced HepG2 Cells

**Figure 8:**
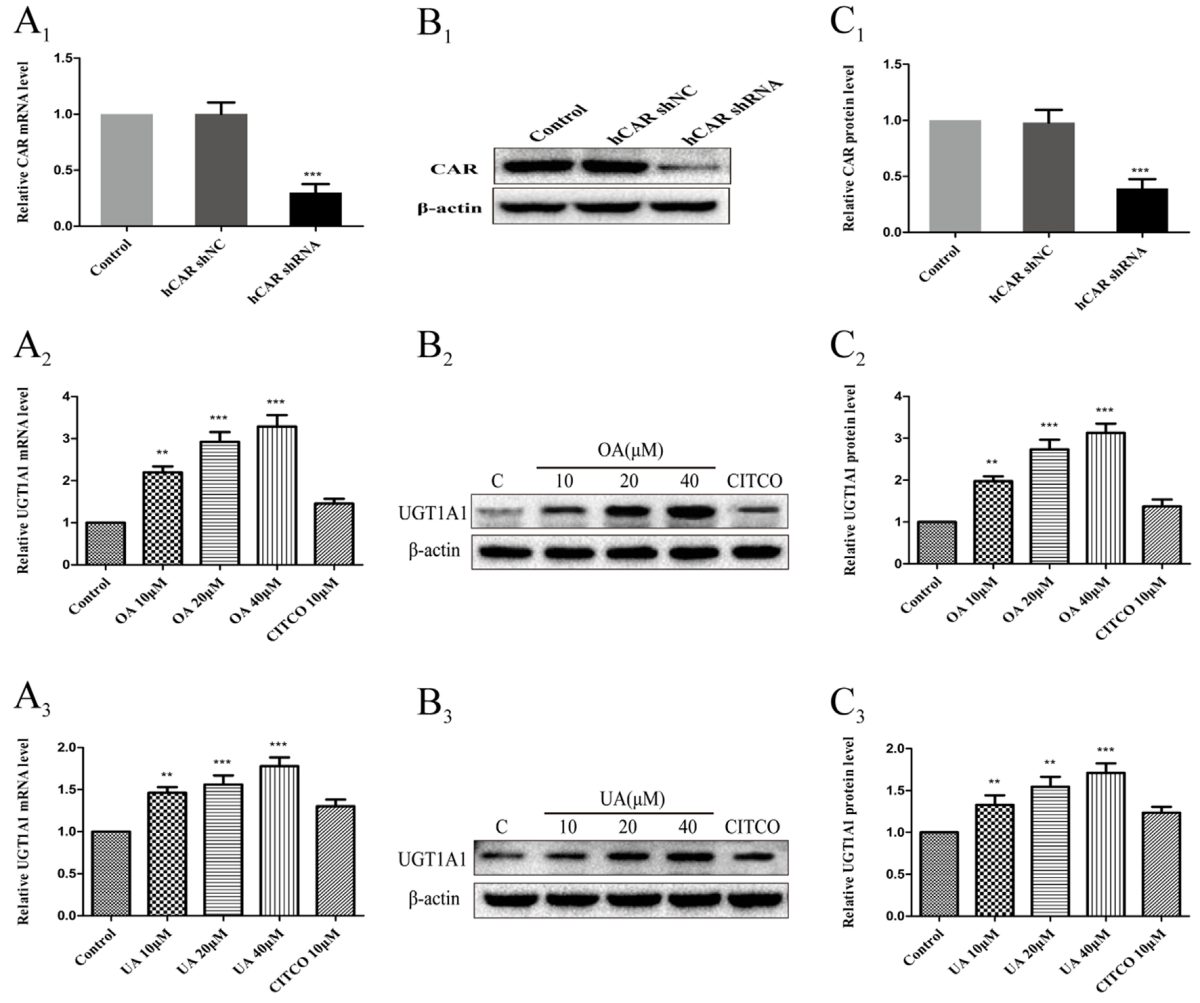
Effect of OA and UA on UGT1A1 mRNA and protein in hCAR-silenced HepG2 cells. CAR mRNA and protein expression in HepG2 cells transfected with hCAR shRNA or hCAR shNC were determined by real-time Q-PCR analysis **(A** **_1_** **)** and Western blotting analysis **(B** **_1_**–**C** **_1_** **)**, respectively. Then the HepG2 cells transfected with hCAR shRNA were treated with OA (10, 20, and 40 μM) **(A** **_2_**–**C** **_2_** **)** and UA (10, 20, and 40 μM) **(A** **_3_**–**C** **_3_** **)** for 24 h. UGT1A1 mRNA and protein expression in hCAR-silenced HepG2 cells treated with OA and UA were determined by real-time Q-PCR analysis **(A)** and Western blotting analysis **(B**–**C)**, respectively. The determination of mRNA and nuclear protein was normalized to GAPDH and β-actin, respectively. DMSO (0.1%) was used as the negative control. CITCO (10 μM) was used as the positive control. The data are expressed as the mean ± SD of triplicate independent experiments (**p < 0.01, ***p < 0.001).

To further confirm whether CAR take part in the regulation of UGT1A1 in HepG2 cells treated with OA (10, 20, and 40 μM) and UA (10, 20, and 40 μM), we successfully constructed an hCAR-silenced HepG2 cell model. Compared to the control group, the expression of CAR mRNA in hCAR-silenced HepG2 cells was downregulated by 67% (**Figure 8A1**), and CAR protein was also markedly reduced (**Figures 8B1, C1**).

Next, UGT1A1 mRNA and protein expression in hCAR-silenced HepG2 cells treated with OA (10, 20, and 40 μM) and UA (10, 20, and 40 μM) were detected by real-time PCR and Western blotting, respectively. Compared to the blank control that was treated with DMSO, UGT1A1 mRNA and protein levels in hCAR-silenced HepG2 cells were significantly elevated when treated with OA (10, 20, and 40 μM) (**Figures 8A2–C2**) and UA (10, 20, and 40 μM) (**Figures 8A3–C3**). However, the UGT1A1 mRNA and protein expression in hCAR-silenced HepG2 cells treated with OA (10, 20, and 40 μM) and UA (10, 20, and 40 μM) did not significantly change compared to that in non-silenced HepG2 cells. The inductive effect of CITCO (10 μM) on UGT1A1 mRNA and protein expression in hCAR-silenced HepG2 cells was significantly lower than that in normal HepG2 cells (**Figures 8A2–C2** and **Figures 8A3–C3**). These results indicated that OA and UA had no influence on the UGT1A1 target gene mediated by CAR.

## Discussion

Several studies have shown that nuclear receptors, especially PXR and CAR, play an important role in the regulation of UGTs, which are involved in the metabolism of various clinical drugs (Tolson and Wang, 2010). UGT1As are highly expressed in the liver and extrahepatic tissues, including UGT1A1, UGT1A3, UGT1A4, UGT1A6, UGT1A7, UGT1A8, UGT1A9, and UGT1A10 (Tukey and Strassburg, 2000; Nakamura et al., 2008). In our study, it was found that the concentrations of UA and OA ranged from 10–40 μM were all non-cytotoxic to HepG2 cells by MTT assay (data not shown). The subsequent studies showed that OA (10, 20, and 40 μM) and UA (10, 20, and 40 μM) significantly upregulated the expression of UGT1A1, UGT1A3, UGT1A4, and UGT1A9 mRNA and protein in HepG2 cells. These results indicated both OA and UA exhibited strong induction on UGT1A1, UGT1A3, UGT1A4, and UGT1A9, and there could be potential drug–drug interactions when OA and UA are co-administered with the substrate drugs of UGT1As. Similar inductive effects of OA and UA on UGT1As may be attributed to the fact that OA and UA are isomers and have similar chemical structures and activities (Kashyap et al., 2016). Seow and Lau (2017) also found UA activated PXR and increased UGT1A3 mRNA expression in human HepG2 and LS180 cells. Furthermore, the effect of UGT1As induced by OA and UA coincided at the mRNA and protein levels, which suggested that OA and UA induced the basal expression of UGT1As mRNA through transcriptional activation and then induced the expression of protein.

Several studies have confirmed that the activity of UGT1A1 is closely related to CAR or PXR, and UGT1A1 is involved in the metabolism of many drugs and important endogenous substances (Sugatani et al., 2005; Yeh et al., 2016). Therefore, we selected UGT1A1 as the representative of UGTs in the following study to explore how OA and UA regulate UGT1As based on the PXR and CAR signaling pathway. In this study, we found that both OA (10, 20, and 40 μM) and UA (10, 20, and 40 μM) significantly increased the expression of PXR mRNA and protein in HepG2 cells in a concentration-dependent manner compared to the control group. However, both OA (10, 20, and 40 μM) and UA (10, 20, and 40 μM) showed no significant impact on CAR mRNA and protein levels in HepG2 cells. In addition, the induction trend of UA and OA on UGT1A1 was in line with that on PXR. Based on the above results, we conclude that the induction of UGT1A1 by OA and UA may be mediated by activating PXR rather than CAR.

To verify the above hypotheses, we further investigated whether OA and UA could induce the PXR/CAR-mediated transactivation of the UGT1A1 by the dual-luciferase reporter assay system. The 290-bp element as a multi-component enhancer is a complex array of transcription factor-binding sites that contain a DR4 element, CAR response element (CARE), DR3 element, and PXR response element (PXRE) (Xie et al., 2003). In the reporter gene assay, the finding that RIF induced the PXR-mediated transactivation of the UGT1A1 290-bp reporter gene (**Figures 5A, B**) supports the fact that RIF is a known inducer of UGT1A1 both *in vitro* (Jemnitz et al., 2002) and *in vivo* (Adachi et al., 1985). Similarly, CITCO induced the CAR-mediated transactivation of the UGT1A1 290-bp reporter gene (**Figures 6A, B**). Meanwhile, both OA (10, 20, and 40 μM) and UA (10, 20, and 40 μM) could induce the PXR-mediated transactivation of the UGT1A1 290-bp reporter gene in a concentration-dependent manner compared to the control group. However, there were no significant impacts on CAR-mediated transactivation of UGT1A1 290-bp reporter gene in HepG2 cells treated with OA (10, 20, and 40 μM) and UA (10, 20, and 40 μM), respectively. These experimental data further supported that both OA and UA can induce UGT1A1 by activating PXR rather than CAR.

To further validate the above hypothesis, an RNA interference experiment was conducted in our study. We observed that the activation efficiency of the UGT1A1 gene in hPXR-silenced HepG2 cells treated with OA (10, 20, and 40 μM) and UA (10, 20, and 40 μM) were significantly reduced compared to the non-silenced HepG2 cells. Knocking down PXR expression by shRNA nearly abolished the induction of OA and UA on UGT1A1. We concluded that PXR activation is critical for the induction of UA and OA on UGT1A1. However, there were no significant differences in the activation efficiency of UGT1A1 in hCAR-silenced HepG2 cells treated with OA (10, 20, and 40 μM) and UA (10, 20, and 40 μM) compared to non-silenced HepG2 cells. Knocking down CAR expression by shRNA did not affect the effect of OA and UA on the UGT1A1 gene. The presence or absence of CAR has no influence on the activation efficiency of UGT1A1 by OA and UA. Therefore, we concluded that OA and UA induce UGT1A1 by activating PXR rather than CAR.

Previous studies have discovered that the UGT1A1 gene contained both CAR and PXR-binging motifs in its promoter region (Maglich et al., 2002; Xie et al., 2003). Among nuclear receptors, PXR and CAR exhibit promiscuous xenobiotic activation capability. Hiura et al. (2014) found that 3-hydroxyflavone can induce UGT1A1 expression *via* PXR in LS180 cells. Roscovitine inhibits phosphorylation of PXR at Ser350 by CDK2 and enhances the translocation of PXR from the cytoplasm to the nucleus, resulting in elevated expression of UGT1A1 (Sugatani et al., 2012). However, phenobarbital treatment for hyperbilirubinemia has been known to increase the expression of the UGT1A1 gene by activating CAR in the liver and show an activity to a 290-bp distal enhancer sequence (−3483/−3194) of the human UGT1A1 gene (Sugatani et al., 2001). Recent studies have demonstrated that PXR and CAR regulate overlapping but distinct sets of genes involved in xenobiotic detoxification (Maglich et al., 2002). Compared to other NRs, PXR possesses a bulky and flexible ligand-binding cavity, which enables it to accommodate a more structurally promiscuous library of ligands (Watkins and Redinbo, 2001; Xu et al., 2004). This supports our conclusion that OA and UA induce UGT1A1 expression by activating PXR rather than CAR. The differential effect mechanism of UA and OA on PXR and CAR is worthy of further study in our future work.

Some previous studies (Kim et al., 2004; Chang et al., 2017) have suggested the links between cytochrome p450 enzymes and OA and/or UA. The metabolites of OA and/or UA mediated by CYP450s may also cause the change (suppression or induction) of UGTs. However, some CYP450 isoenzymes are hardly expressed in HepG2 cells such as CYP2E1. Therefore, it is needed to investigate the effect of UA and OA on UGTs using human primary hepatocytes in the incoming study.

In summary, OA and UA can induce the expression of UGT1A1, UGT1A3, UGT1A4, and UGT1A9 in HepG2 cells, and there are some potential drug–drug interactions when OA and UA are co-administered with clinical drugs of UGT1As substrates. OA and UA induced UGT1A1 expression by activating PXR rather than CAR. Additional studies are needed to assess the physiological significance of OA- and UA-activated PXR in animal models.

## Data Availability Statement

The raw data supporting the conclusions of this manuscript will be made available by the authors, without undue reservation, to any qualified researcher.

## Author Contributions

Participated in research design: CX, CZ and YX. Conducted experiments: NY, TZ, CZ, FH and ML. Performed data analysis: NY, FL, HZ and CX. Wrote or contributed to the writing of the manuscript: NY and CX.

## Conflict of Interest

The authors declare that the research was conducted in the absence of any commercial or financial relationships that could be construed as a potential conflict of interest.

## Funding

This work was supported by China National Nature Science Fund (Nos. 81560606 and 81760672).

## Abbreviations

OA, oleanolic acid; UA, ursolic acid; PXR, pregnane X receptor; CAR, constitutive androstane receptor; UGTs, UDP-glucuronosyltransferases; UGT1A1, UDP-glucuronosyltransferase 1A1; Q-PCR, quantitative real-time polymerase chain reaction analysis; SN-38, 7-Ethyl-10-hydroxycamptothecin; RXR, retinoid X receptor; RIF, rifampicin; CITCO, Six-(4-chlorophenyl)imidazo (2,1-b)(1,3) thiazole-5-carbaldehydeO-(3,4-dichlorobenzyl)oxime; DMSO, dimethyl sulfoxide; DMEM, Dulbecco’s modified Eagle’s Medium; FBS, fetal bovine serum; BCA, bicinchoninic acid assay; hCAR shRNA, shRNA against human CAR; hPXR shRNA, shRNA against human PXR; shNC, negative control non-silencing RNA.
